# Topology constrained nonnegative matrix factorization for time varying omic expression

**DOI:** 10.1038/s41598-026-43968-w

**Published:** 2026-03-12

**Authors:** Anirban Dey, Kaushik Das Sharma, Amitava Chatterjee, Pritha Bhattacharjee

**Affiliations:** 1 Institute of Technical Education & Research, Siksha ‘O’ Anusandhan, Bhubaneswar, India; 2 Department of Applied Physics, University of Calcutta, Kolkata, India; 3 Department of Electrical Engineering, Jadavpur University, Kolkata, India; 4 Department of Environmental Science, University of Calcutta, Kolkata, India

**Keywords:** Nonnegative matrix factorization, ‘Omic profiles, Feature representational learning, Type-2 diabetes, Huntington disease, Computational biology and bioinformatics, Systems biology

## Abstract

Deciphering disease-specific progression from low sample size, high-dimensional omic profiles remains challenging. Traditional biomarker discovery methods are costly and limited, while Nonnegative Matrix Factorization (NMF), though popular, suffers from instability and lack of biologically relevant solutions. This study aims to overcome these limitations by introducing a more robust framework. This article proposes *TopConNMF*, a topology-constrained extension of NMF which incorporates structural constraints, ensures stability, accuracy, and faster performance while maintaining biological interpretability. The method was evaluated on two publicly available time-varying omic datasets with established ground truths and compared against other *state-of-the-ar*t approaches. The *TopConNMF* consistently demonstrated stable performance across both the datasets, delivering superior accuracy and biologically relevant factorization compared to conventional NMF and other benchmark methods. The exhaustive evaluation confirmed its robustness in capturing disease-specific profiles and its efficiency in handling complex, high-dimensional data. Thus, *TopConNMF* provides a deeper understanding of complex biological systems by producing stable and interpretable factorization. Its broad applicability across multiple disease manifestations highlights its potential as a valuable tool for advancing omic data analysis and biomarker discovery. ***Clinical Impact***: *TopConNMF* enables reliable biomarker discovery from limited omic data, supporting early diagnosis, patient stratification, and personalized treatment, thereby bridging computational findings with clinical applications.

## Introduction

This conditions can disrupt everyday life and activities, with certain complex conditions presenting significant health challenges^1^. These conditions are often prevalent, recurrent, and have a tendency to spread, necessitating ongoing research into their mechanisms. For instance, a common health condition related to lifestyle choices can lead to various subtypes, each requiring different treatment strategies^2^. Resistance to treatment can occur, highlighting the need for precision medicine through the identification of specific biomarkers^1^. These markers are crucial for developing targeted therapies, which require a deep understanding of the molecular processes to identify the most effective treatment targets^3^.

Several extensive studies are conducted on finding these biological markers^4–7^. Although, the traditional methods of analysis may not always provide a complete understanding of the underlying causes of the condition^4,7^. The use of diverse data types, beyond just one, can offer greater insights into the intricate biological network of toxicity. This includes the study of various changes in the ‘omic space, all of which contribute to our understanding of ‘omic expression and its role in disease development^5,7^.

In addressing these challenges, the feature extraction and feature selection are the most prominent approaches. It has been observed that in past two decades that the feature selection methods has gained its prominence particularly due to their effectiveness in terms of interpretability while understanding the most informative biomarkers^8,9^. In order to selecting a small set of biomarkers from vast ‘omic datasets, the feature selection has a superior computational efficiency as well as a streamlined reasonable approach in selecting the biomarkers^10^. Therefore, this technique offers a substantial advantages to serves as a pre-processing step to identify the most fundamental biomarkers that boost classification performance^8,9^. Further, based on data label availability these feature representational learning methods can be broadly divided into three main categories namely: supervised, unsupervised, and semi-supervised^8^. In case of supervised and unsupervised methods ‘omic profiles are either fully labeled or fully not labeled respectively.

**Fig. 1:**
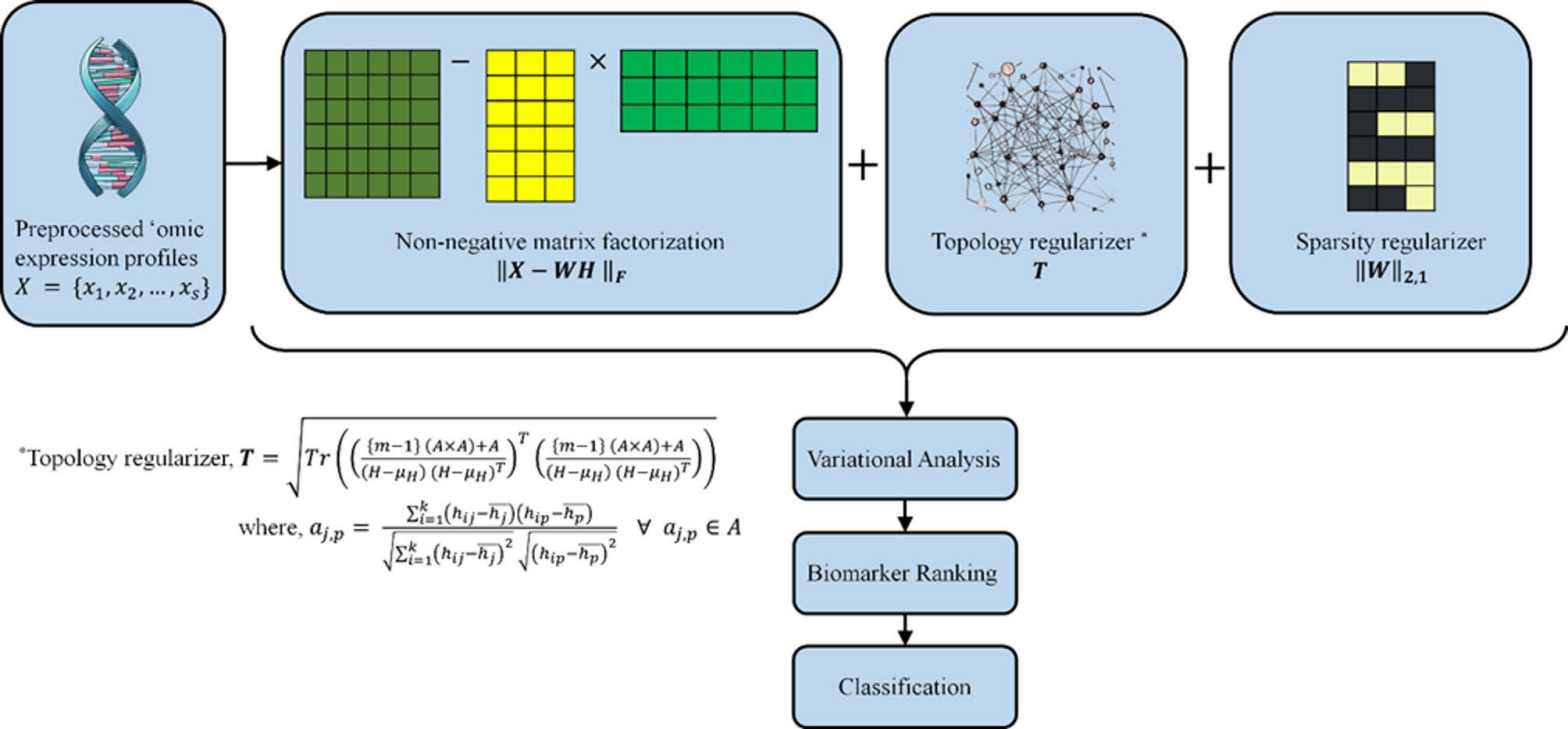
Schematic representation of the proposed *TopConNMF*.

On the other hand, the semi-supervised methods leverage a mix of labeled and unlabeled data to enhance the efficacy of unsupervised approaches^8,9^. Again, based on the operational approach the feature selection methods are categorized into two main categories, namely, filter and wrapper. The Filter methods identify relevant features without depending on learner. On the contrary the wrapper methods evaluate the importance of a biomarker in an online fashion based on the predictive performance pf the learner^11^. There is another approach that has been into the lime light which embeds a filter methods on top of the wrapper. This method is sometimes referred as ensemble method. Therefore, in the realm of unsupervised feature selection, as a representational learning paradigm the absence of labeled data presents a greater challenge than in supervised or semi-supervised scenarios, as it cannot benefit from pre-existing label information. Thus, this results in a developing a model which is generalizable as well as less prone to overfitting. Now, in this scenario, the most critical part is to infer the intrinsic geometric structure of the data, which is crucial for effective feature selection without labels^12^.

Recent developments in machine learning have introduced advanced bioinformatics frameworks that leverage deep feature embedding and adaptive graph learning to improve omic data interpretability^13–15^. These studies collectively emphasize the role of structural regularization and topology-aware learning in enhancing biomarker discovery. Building upon these insights, the present work extends conventional graph-regularized NMF by integrating a topology-overlap regularizer that captures both direct and indirect molecular associations, thereby ensuring more biologically meaningful factorization.

Now, to address the above stated issue in this genera of representational learning paradigm, Nie et al.^16^ presented a technique that merges linear regression with manifold regularization to produce pseudo-labels, laying the groundwork for both semi-supervised and unsupervised feature selection systems. This research^16^ has given rise to two primary schools of thought. The first, known as the *Sparse Regression* approach^17,18^, leverages sparse regression models to generate the pseudo-labels. On the other hand the second approach known as *Spectral Regression and Adaptive Graph Learning*, employs spectral regression to understand the local manifold structure of the data during feature selection^19^. The recent development in this field introduced a dual regularized subspace learning as well as adaptive graph learning method together for unsupervised feature selection to tackle the issue of imbalanced neighbors^12,20^. It is also crucial to note that most of these methods typically depends on kernel functions within the feature space which are often susceptible to noise, resulting to a compromise its reliability^21^.

Therefore, considering the challenges in our hand, this paper proposes an unsupervised filtering approach as depicted in Fig. 1 to select data driven biologically relevant biomarkers from a time varying ‘omic data. This method uses Nonnegative matrix factorization with constrained topological and sparsity regularization. The method involves the topological regularizer applied to the NMF where interactions between genes or proteins are captured. It is because the connections between these biological entities often indicate similar functions or involvement in the same biological process. However, the data representing these connections may not always be flawless, necessitating methods to understand their relationships. Secondly, the ${L}_{\mathrm{2,1}}$-norm is employed to estimate the sparsity thus the low-rank constraint of the basis. These constraints will enhance the preservation of local information while enforcing sparsity, which in-turn enable us to incorporate of significant sub-groups from the biological network, thus leads to the discovery of inherent patterns in the ‘omic data during the process of biomarker identification.

Further the major contributions of this work could be summarized as:

- A Relaxed Nonnegative matrix factorization based approach has been proposed to incorporate the regulatory effects in the ‘omic data.
- The approach also embodies an adaptive learning structure that helps to preserve the topological properties of the data.
- Finally, the formulated dual regularized feature representational learning paradigm is capable of capturing an optimal/near-optimal differentially expressing ‘omic biomarkers.

The rest of this paper is structured as follows: Section II presents a concise overview of NMF based unsupervised feature selection methods. The proposal is elaborated in Section III. Section IV presents the experimental outcomes that demonstrates the efficacy of the proposed method. Finally, Section V concisely concludes this research.

### Preliminaries

This section presents an overview of the evolution of feature selection techniques in nonnegative matrix factorization (NMF), a method used since the 1970s^22^. Although, after a significant advancement in 2015^23^ where NMF was used to select features by decomposing the data matrix into two smaller, nonnegative matrices, $W$and$H$. The goal was to minimize the error between the original matrix ($X$) and the reconstructed matrix ($\stackrel{\sim}{X}$) (i.e.$\stackrel{\sim}{X}=WH$), under a specific orthogonality condition (i.e.$W{W}^{T}=I$). This primary motive of this work was to establish an indicator matrix to rank features, but later it was found that this orthogonality condition is not sufficient enough to make an indicator matrix^24^. Again, Jiang et al.^25^ introduced a regularized version of NMF that uses a Laplacian graph and a special matrix norm (${L}_{\mathrm{2,1}}-Norm$) to preserve local information and ensure sparsity, leading to better feature selection. This regularized NMF used an adaptive structure learning and a unique regularization approach, which refined the Laplacian matrix and its sparsity^25^. Similarly, Wang et al. in^26^ not only considers sparsity and structural learning but also incorporates a rank constraint to leverage inherent low-rank characteristics of the data^26^. This method employs a Laplacian graph for local preservation, a specific matrix norm for sparsity, and utilizes Ky Fan’s theorem to determine the decomposition rank by the sum of the smallest singular values^26^.

It is evident from the *state-of-the-arts* that the use of NMF in bioinformatics is primarily to break down expression data into components (i.e. the basis and the coefficient metrics) that represents the population in reduced space while keeping the influence of ‘omic regulation under consideration^11,25,27^. It has always been critical to understand the disease manifestation by the means of finding the most biologically relevant differentially expressing biomarkers that are capable of differentiating exposed and control populations based on their genetic expression patterns^11,25,27^. Therefore, motivated by this, Wang et al. in^26^ have used an approach to perform an unsupervised cluster analysis to identify genomic markers in single-cell RNA sequencing data to determine cell types. The joint NMF meta-analysis (*j*NMFMA)^26^, has been effective in finding the genes linked to diseases. Thus, the method utilize the concept of concurrently break down several transcriptomics data into a single shared submatrix and multiple distinct submatrices. Further, Jiang et al.^25^ introduced flexible NMF (FNMF), which relaxes the positive element constrain (i.e.$W\in{\mathbb{R}}_{\pm}^{g\times{k}}$) and an adaptable to sparsity constrain by the means of${L}_{\mathrm{2,1}}-\mathrm{N}\mathrm{o}\mathrm{r}\mathrm{m}$ to account for regulatory influences. It is observed that none of the work focuses on isolating biomarkers and tracking the progression of diseases. Therefore, to address the major limitations, in our previous research^11^ we introduced a multi-level self-organizing map (MLSOM), which yielded encouraging outcomes.

Although, building upon this groundwork, it is also observed that mostly research has improved the challenges associated with selecting features using NMF. However, there are two main issues that remains still unaddressed. Firstly, regularization is typically applied only to the feature space. Secondly, the structural constrained applied on the data always assumed to be constant. Therefore, to address these issues, this paper introduces an approach that uses dual regularization leverages both the feature weight and representation matrices, as well as the data’s natural low-rank structure, to enhance sparsity.

## Method

This section describes the proposed method for selecting the ‘omic biomarkers from a time varying profiles by formulating a convex optimization problem and develop an update rule to solve it. This method is designed with a two-part objective function that includes a cost function for data subspace, a regularization to maintain the topology, a sparsity regularization to highlight important features and improve feature set quality.

### Subspace Learning

Feature selection is a process to effectively represents the entire set of ‘omic data in terms smaller set of biomarker. To illustrate, imagine a complete set of biomarkers$X=\{{\mathcal{g}}_{1},{\mathcal{g}}_{2},\dots,{\mathcal{g}}_{s}\}$. From this, a smaller subset containing$k$biomarkers,$W=\{{\mathcal{g}}_{1},{\mathcal{g}}_{2},\dots,{\mathcal{g}}_{k}\}$ such that$k\ll{s}$. This subset,$W$ is actually part of the larger matrix$X$, (i.e.$W\subset{X}$). Thus, the goal of feature selection is to ensure that $\stackrel{\sim}{X}=WH$ closely approximates the full feature set$X$. Therefore, the concept could be quantified through a minimization problem that minimizes the subspace distance, mathematically defined as:

1 $$\begin{array}{c}\mathrm{a}\mathrm{r}\mathrm{g}\mathrm{m}\mathrm{i}\mathrm{n}\\W\end{array}\overrightarrow{{S}_{d}}\left(span\left(X\right),span\left(W\right)\right):\left|G\right|=k$$

where, $\overrightarrow{{S}_{d}}$ is the subspace distance function, $k$ is dimension of the reduced feature space, and $G$ is a set of selected genes. In the context of nonnegative matrix factorization, feature selection is further refined through subspace learning, which is defined by the following equation:

2 $$\begin{array}{c}\mathrm{a}\mathrm{r}\mathrm{g}\mathrm{m}\mathrm{i}\mathrm{n}\\W\end{array}\overrightarrow{{S}_{d}}\left(span\left(X\right),span\left(W\right)\right)=\genfrac{}{}{0pt}{}{\mathrm{m}\mathrm{i}\mathrm{n}}{W}{\parallel{X}-WH\parallel}_{F}$$

where, $H$ represents the coefficient matrix that holds the mixing coefficient vectors of the original feature space for the selected basis ($W$). In other words, $W$ is the lower-dimensional representation of$X$, achieved by projecting $X$ through a projection matrix ($H$). This matrix, $H\in{\mathbb{R}}_{\pm}^{k\times{s}}$could be seen as:

3 $${h}_{i,j}=\left\{\begin{array}{c}1if{x}_{j}\in{X}fullyrepresents{w}_{i}\\0otherwise\end{array}\right.$$

This mathematical framework allows for the selection of a feature subset that maintains the integrity of the ‘omic data while reducing its complexity. Thus, the subspace of $W$ is therefore defined as:

4 $$W=X{H}^{T}$$

The process of selecting features is therefore could be framed as a minimization problem, defined as:

5 $$\mathrm{min}{\parallel{X}-WH\parallel}_{F}\mathrm{S}\mathrm{u}\mathrm{b}\mathrm{j}\mathrm{e}\mathrm{c}\mathrm{t}\mathrm{t}\mathrm{o}:W\ge0\mathrm{a}\mathrm{n}\mathrm{d}H\ge0$$

It is already established that the non-negativity and orthogonality conditions introduced in^28^ are alone insufficient conditions. Thus, overcome this, the introduction of sparse regularization methods, including${L}_{\mathrm{2,1}}-\mathrm{N}\mathrm{o}\mathrm{r}\mathrm{m}$,${L}_{2,p}-\mathrm{N}\mathrm{o}\mathrm{r}\mathrm{m}$, inner product norm, etc. has been suggested^29,30^. While traditionally, regularization has been applied solely to the feature weight matrix $W$^29,30^, the *state-of-the-art* advocates for the regularization of both the coefficient ($H$) and basis ($W$) matrix to enhance the quality of biomarker selection^11,25,31^. This motivated us to introduce a dual regularization approach for both matrices $W$ and$H$. Therefore, it is adopted in this study. Consequently, the minimization problem in (5) is updated as:

$$\mathrm{Min}{\parallel{X}-WH\parallel}_{F}+\alpha{Reg}\left(W\right)+\beta{Reg}\left(H\right)$$

6 $$\mathrm{S}\mathrm{u}\mathrm{b}\mathrm{j}\mathrm{e}\mathrm{c}\mathrm{t}\mathrm{t}\mathrm{o}:W\ge0\mathrm{a}\mathrm{n}\mathrm{d}H\ge0$$

where, $Reg\left(W\right)$and $Reg\left(H\right)$ are the regularization terms associated with the basis ($W$) and coefficients ($H$) respectively. The parameters $\alpha$and$\beta$are the regularization coefficients and these selected depending upon the problem in hand.

### Problem formulation

Now, in order to formulate the cost, we need to first define the basis and coefficient regularization as presented in (6), for a better understanding the problem.

#### Basis regularizer

A variety of sparse regularization functions have recently seen to be gaining a lot of attention^29,30^. It is seen that the ${L}_{\mathrm{2,0}}-$Norm is an optimal regularizer for producing the sparsest solution. Although, it is non-convex and leads to an NP-hard problem^12,30^. The ${L}_{\mathrm{2,1}}-$Norm is another common approximation for the ${L}_{\mathrm{2,0}}-$Norm and is frequently used for sparse regularization^32^. However, in some cases the ${L}_{2,p}-$Norm for $0<p<1$ can yield sparser solutions than the${L}_{\mathrm{2,1}}-$Norm but though it enhance sparsity on the basis vectors${w}_{j}\in{W}$, it is not a real norm as it does not satisfy the triangle inequality^12^. Experimental results indicate that the ${L}_{2,p}-$Norm can achieve the lowest classification error compared to the ${L}_{\mathrm{2,1}}-$Norm but neither convex nor Lipschitz continuous^12,32^, which again significantly reduces the control on the gradient in each iteration.

Thus, in this paper, we have focused on ${L}_{\mathrm{2,1}}-$Norm as the regularization favors for its ability to induce sparsity in solutions and its robustness to outliers, unlike the other regularizes including inner product norm which associated with orthogonality and certain mathematical properties as proposed in^12,25^. It is noteworthy to mention that the choice of using this regularizer is solely for the desired sparsity, robustness to outliers, and relevance of orthogonality to the problem in hand. While the other norm offers many valuable properties, but because of the above stated reasons the ${L}_{\mathrm{2,1}}-$Norm is preferred in this scenarios. Hence, the regularization for the basis matrix, $W$ is formulated as follows:

7 $$Reg\left(W\right)={\parallel{W}\parallel}_{\mathrm{2,1}}={\sum}_{i=1}^{g}\sqrt{\sum_{j=1}^{k}{w}_{i,j}^{2}}$$

#### Coefficient regularizer (topology regularization)

The cost in (5) for the matrix factorization is primarily to reduce the reconstruction error between $X$ and the product $\stackrel{\sim}{X}=WH$ and this is achieved by approximating each original data, and is mathematically defined as:

8 $$\stackrel{\sim}{X}=\left[{\mathcal{g}}_{1},{\mathcal{g}}_{2},\dots,{\mathcal{g}}_{s}\right]=WH$$

Therefore, individual biomarkers ${\mathcal{g}}_{i}\in\stackrel{\sim}{X}\forall{i}=\{\mathrm{1,2},\dots,s\}$ is composition of the coefficient vectors and the basis matrix, mathematically could be visualized as:

9 $$\stackrel{\sim}{X}=\left\{\begin{array}{c}{\mathcal{g}}_{1}\approx{W}\times{h}_{1}\\\vdots\\{\mathcal{g}}_{s}\approx{W}\times{h}_{s}\end{array}\right.$$

where, $W\in{\mathbb{R}}_{+}^{g\times{k}}$and${h}_{i}\forall{i}=\{\mathrm{1,2},\dots,s\}$are the column vectors. The relationship in (9), where ${h}_{i}$ represents the column vector of the coefficient matrix$H$. This implies that any given biomarker ${\mathcal{g}}_{i}$ can be approximated as a linear combination of the basis matrix and the coefficient vector. Moreover, it is also evident that there is a tangible connection between ${\mathcal{g}}_{i}$ and the column vector${h}_{i}$. This, leads to the conclusion that Eq. (9) demonstrates the self-representation characteristic of the biomarkers. Thus, underscores the indispensable influence of $H$ on this self-representation aspect of the chosen${\mathcal{}\mathcal{g}}_{i}\in\stackrel{\sim}{X}\forall{i}=\{\mathrm{1,2},\dots,s\}$. In simpler terms, if two biomarkers ${\mathcal{g}}_{i}$ and ${\mathcal{g}}_{j}$ are unrelated, their corresponding columns vectors, ${h}_{i}$ and${h}_{j}$, should also be unrelated. The degree of correlation between the columns of $H$ is indicative of the correlation between themselves. So, to emphasize the significance of the matrix $H$ in the process of feature selection, it is essential to recognize that $H$ not only captures the linear relationships but also the complex, nonlinear interactions that may exist between biomarkers.

Therefore, inspired by topological overlap matrix (TOM) proposed by Andy et al.^33^ we may say that the coefficient matrix$H$, holds a comprehensive behavioral representation of the biomarkers’ that reflects both direct and indirect associations. This comprehensive view is crucial for accurate feature selection, as it ensures that the interconnectedness of biomarkers is fully accounted for, leading to more reliable and valid biomarker identification. Therefore, the interconnectedness could be defined by the correlation matrix$A$, is defined as:

10 $${a}_{j,p}=\frac{{\sum}_{i=1}^{k}\left({h}_{ij}-\stackrel{-}{{h}_{j}}\right)\left({h}_{ip}-\stackrel{-}{{h}_{p}}\right)}{\sqrt{{\sum}_{i=1}^{k}{\left({h}_{ij}-\stackrel{-}{{h}_{j}}\right)}^{2}}\sqrt{{\left({h}_{ip}-\stackrel{-}{{h}_{p}}\right)}^{2}}}\forall{a}_{j,p}\in{A}$$

Again, the robustness of $H$ in capturing the topological essence of the data is evident when considering the stability of feature selection. In the presence of noise or when dealing with high-dimensional data, with low sample size, the ability of $H$ to find the patterns of true associations from spurious pattern is invaluable. By leveraging the shared neighbor’s concept inherent in the topological overlap measure, $H$ can mitigate the effects of noise and reduce the likelihood of false positives in the feature selection process. Thus, the topological constrained could be defined as:

11 $$\boldsymbol{T}=\sqrt{Tr\left({\left(\frac{\left\{k-1\right\}\left(A\times{A}\right)+A}{\left(H-{\mu}_{H}\right){(H-{\mu}_{H})}^{T}}\right)}^{T}\left(\frac{\left\{k-1\right\}\left(A\times{A}\right)+A}{\left(H-{\mu}_{H}\right){(H-{\mu}_{H})}^{T}}\right)\right)}$$

where, $k$ is the factorization rank of the NMF, ${\mu}_{H}$ is the mean vector of the coefficient matrix ($H$). Therefore, $H$ embodies the essence of topological structure, providing a robust and sensitive framework for the analysis of ‘omic data.

Unlike conventional graph-regularized NMF methods that rely solely on Laplacian or correlation-based constraints, the proposed topology-overlap regularizer in *TopConNMF* is derived from the Topological Overlap Matrix (TOM)^29^, which quantifies both direct and indirect associations among biomolecules through shared neighbors^15,34^. This formulation extends beyond traditional Laplacian regularization by capturing higher-order biological connectivity patterns rather than only pairwise similarities^14^. By integrating this topology-aware constraint with the sparsity-inducing ${L}_{\mathrm{2,1}}-$norm, *TopConNMF* jointly enforces local manifold smoothness and global topological coherence within the factorization space^35^. The resulting gradient formulation enables efficient multiplicative updates while preserving the intrinsic biological topology in the learned representation, thereby producing more interpretable and noise-resilient decompositions of time-varying omic data. This dual regularization mechanism distinguishes TopConNMF from previous state-of-the-art approaches^11,22,23^.

#### Cost function

The cost for the proposed *TopConNMF* can be now be formulated by integrating regularizer in (7) and (11) into the problem in (6) as:

$$\mathrm{Min\:}\mathcal{J}\left({\uptheta}\right)={\parallel{X}-WH\parallel}_{F}+\alpha{\parallel{W}\parallel}_{\mathrm{2,1}}+\beta{T}$$

12a $$\mathrm{S}\mathrm{u}\mathrm{b}\mathrm{j}\mathrm{e}\mathrm{c}\mathrm{t}\mathrm{t}\mathrm{o}:W\ge0\mathrm{a}\mathrm{n}\mathrm{d}H\ge0$$

Further, (12a) could be rewritten as:

$$\mathrm{Min\:}\mathcal{J}\left({\uptheta}\right)={\parallel{X}-WH\parallel}_{F}+\alpha{\sum}_{i=1}^{g}\sqrt{\sum_{j=1}^{k}{W}_{i,j}^{2}}+\beta\sqrt{Tr\left({\left(\frac{\left\{k-1\right\}\left(A\times{A}\right)+A}{\left(H-{\mu}_{H}\right){\left(H-{\mu}_{H}\right)}^{T}}\right)}^{T}\left(\frac{\left\{k-1\right\}\left(A\times{A}\right)+A}{\left(H-{\mu}_{H}\right){\left(H-{\mu}_{H}\right)}^{T}}\right)\right)}$$

12b $$\mathrm{S}\mathrm{u}\mathrm{b}\mathrm{j}\mathrm{e}\mathrm{c}\mathrm{t}\mathrm{t}\mathrm{o}:W\ge0\mathrm{a}\mathrm{n}\mathrm{d}H\ge0$$

#### Update rules

In the context of cost function in (12b), the variables $W$ and $H$ are updated through specific rules as the function presents a non-convex nature^28^. Thus, making it a complex challenge for direct resolution. Although, an iterative method could be applied where, $W$ or $H$ could be updated individually while fixing the other^11,25^. To ensure the non-negativity constrained (i.e.$W\ge0$,$H\ge0$) is imposed on the cost, two Lagrange multipliers are utilized. Therefore, the Lagrangian of the cost function in (12b) could be written as:

13 $$\begin{aligned}\mathcal{L}=Tr\left(X{X}^{T}\right)+2Tr\left(XW{H}^{T}\right)\\+2Tr\left(W{H}^{T}H{W}^{T}\right)\\+\alpha{Tr}\left({{(W}^{T}W)}^{\raisebox{1ex}{$1$}\!\left/\!\raisebox{-1ex}{$2$}\right.}\right)+\beta\sqrt{Tr\left({\left(\frac{\left\{k-1\right\}\left(A\times{A}\right)+A}{\left(H-{\mu}_{H}\right){\left(H-{\mu}_{H}\right)}^{T}}\right)}^{T}\left(\frac{\left\{k-1\right\}\left(A\times{A}\right)+A}{\left(H-{\mu}_{H}\right){\left(H-{\mu}_{H}\right)}^{T}}\right)\right)}+Tr\left({\phi}_{1}W\right)+Tr\left({\phi}_{2}H\right)\end{aligned}$$

where, ${\phi}_{1}$ and ${\phi}_{2}$are the Lagrangian multipliers.

Now, to construct the update rule for$W$; we need to compute the gradient of (13) with respect to$W$. Thus, the computed gradient is:

14 $$\partial\mathcal{L}/\partial{W}=-2{X}^{T}H+2W{H}^{T}H+\alpha{W}+{\phi}_{1}$$

Now, using KKT conditions for optimality, and setting ${\phi}_{1}W=0$ we get:

15 $${\left[-2{X}^{T}H+2W{H}^{T}H+\alpha{W}\right]}_{i,j}{W}_{i,j}=0$$

Therefore, the update rule for $W$ may be written as:

16 $${W}_{i,j}\leftarrow{W}_{i,j}\frac{{\left[2{X}^{T}H\right]}_{i,j}}{{\left[2W{H}^{T}H+\alpha{W}\right]}_{i,j}}$$

Similarly, to construct the update rule for$H$; we need to compute the gradient of (13) with respect to$H$. Thus, the computed gradient is:

17 $$\partial\mathcal{L}/\partial{H}=-2{X}^{T}H+2W{H}^{T}H+\beta\partial{T}/\partial{H}+{\phi}_{1}$$

where, $T$ is the topology regularizer defined in (11).

Now, to solve (17), we first need to find${T}^{{\prime}}=\partial{T}/\partial{H}$. In this case we may begin by finding the derivative of the argument of the trace ($Tr$(.)) *w.r.t.* $H$ and then proceed with the chain rule, to find the derivative of (17).

Let, the inner function in (11) be

$$G=B/\left\{\left(H-{\mu}_{H}\right){\left(H-{\mu}_{H}\right)}^{T}\right\}$$

where,

18 $$B=\left(k-1\right)\left(A\times{A}\right)+A$$

Now, the derivative of (18), *w.r.t.* H is:

19 $${G}^{{\prime}}={B}^{T}{\left(H-{\mu}_{H}\right)}^{-T}{\left(H-{\mu}_{H}\right)}^{-1}{B\left(H-{\mu}_{H}\right)}^{-T}{\left(H-{\mu}_{H}\right)}^{-1}{\left(H-{\mu}_{H}\right)}^{-1}-{\left(H-{\mu}_{H}\right)}^{-1}{B}^{T}{\left(H-{\mu}_{H}\right)}^{-T}{\left(H-{\mu}_{H}\right)}^{-1}{\left(H-{\mu}_{H}\right)}^{-1}{B}^{T}$$

Again, (11) in terms of $G$ could be rewritten as:

20 $$T=\sqrt{Tr\left({G}^{T}G\right)}$$

So, the derivative of (20), *w.r.t.* H is:

21 $${T}^{{\prime}}=\partial{T}/\partial{H}=\raisebox{1ex}{$1$}\!\left/\!\raisebox{-1ex}{$2$}\right.\sqrt{G}{G}^{{\prime}}$$

Substituting the value of $G$ and ${G}^{{\prime}}$ form (18) and (19) in (21), we get,

22 $${T}^{{\prime}}=\frac{{B}^{T}{\left(H-{\mu}_{H}\right)}^{-T}{\left(H-{\mu}_{H}\right)}^{-1}{B\left(H-{\mu}_{H}\right)}^{-T}{\left(H-{\mu}_{H}\right)}^{-1}{\left(H-{\mu}_{H}\right)}^{-1}}{\sqrt{Tr\left({\left(\frac{\left\{k-1\right\}\left(A\times{A}\right)+A}{\left(H-{\mu}_{H}\right){\left(H-{\mu}_{H}\right)}^{T}}\right)}^{T}\left(\frac{\left\{k-1\right\}\left(A\times{A}\right)+A}{\left(H-{\mu}_{H}\right){\left(H-{\mu}_{H}\right)}^{T}}\right)\right)}}+\frac{{\left(H-{\mu}_{H}\right)}^{-1}{B}^{T}{\left(H-{\mu}_{H}\right)}^{-T}{\left(H-{\mu}_{H}\right)}^{-1}{\left(H-{\mu}_{H}\right)}^{-1}{B}^{T}}{\sqrt{Tr\left({\left(\frac{\left\{k-1\right\}\left(A\times{A}\right)+A}{\left(H-{\mu}_{H}\right){\left(H-{\mu}_{H}\right)}^{T}}\right)}^{T}\left(\frac{\left\{k-1\right\}\left(A\times{A}\right)+A}{\left(H-{\mu}_{H}\right){\left(H-{\mu}_{H}\right)}^{T}}\right)\right)}}$$

Finally, using KKT conditions for optimality, and setting ${\phi}_{2}H=0$ we get:

23 $${\left[-2{X}^{T}W+2H{W}^{T}H+{T}^{{\prime}}\right]}_{i,j}{H}_{i,j}=0$$

Therefore, the update rule for $W$ may be written as:

24 $${H}_{i,j}\leftarrow{H}_{i,j}\frac{{\left[-2{X}^{T}W\right]}_{i,j}}{{\left[2H{W}^{T}H+\beta{T}^{{\prime}}\right]}_{i,j}}$$

where, ${T}^{{\prime}}$ is already defined in (22).

It is worth noting that the additional term $\beta{T}{\prime}$ is nonnegative and only modifies the denominator scaling in the multiplicative update. Therefore, the non-negativity preservation and convergence properties of the classical NMF update rule remain valid under the proposed formulation.

Thus, using (16) and (24), the basis and the coefficient matrices could be updated iteratively.

#### Numerical stability and conditioning

To ensure numerical stability during the optimization process, a numerical flooring mechanism was applied wherein any undefined or non-finite values ($NaN$ or $\infty$) were replaced with a small constant ($1\times10^{-s}$). This pragmatic stabilization approach prevents computational divergence and maintains smooth convergence without modifying the objective function. Alternatively, in line with standard matrix conditioning practices^14^, a small diagonal perturbation term ($\epsilon{I}$) may also be used to precondition matrix inversions; however, empirical analysis indicated that the flooring method achieved sufficient stability for this study.

#### Variational analysis and biomarker ranking

A hierarchical SOM architecture as discussed in^11^ is used to cluster the coefficient vectors${h}_{j}\forall{j}=\{\mathrm{1,2},\dots,s\}$into vital and trivial biomarkers, with further subdivisions based on their co-expression patterns^11^. The architecture employs a winner-takes-all mechanism for input processing, described as:

25 $$\parallel{h}\left(t\right)-{\omega}_{bmu}\left(t\right)\parallel\le\parallel{h}\left(t\right)-{\omega}_{i}\left(t\right)\parallel\forall{i}$$

26 $${w}_{i}(t+1)={\omega}_{i}\left(t\right)+\eta\left(t\right){N}_{i}\left(t\right)\left(h\right(t)-{\omega}_{i}(t\left)\right)$$

where, ${w}_{i}$are the weight vectors and ${N}_{i}\left(.\right)$ is a neighborhood functions as defined in^11^. The study utilizes a two-layer hierarchy, with $C-$means clustering to assimilate outputs into clusters^11^. These clusters are then processed by subsequent SOM layers, defined as follows:

27 $$d\left(Y,c\right)={\parallel{y}_{i}-\left(\raisebox{1ex}{$1$}\!\left/\!\raisebox{-1ex}{$k$}\right.\left\{{\sum}_{i}{y}_{i}\right\}\right)\parallel}_{2}\forall{i}$$

where, $Y$ is the output of the SOM, and c is the cluster centroid.

Further, the clustered coefficient vectors into four groups are analyzed to determine class belongingness using the following equation^11^:

28 $${\mu}_{B}=\sum_{p=1}^{{n}_{B}}\left({\left|{h}_{B}^{s,p}\right|}_{1}\right),\forall{p}\in\left(s+1,\dots,s+40\right)$$

29 $${\mu}_{D}=\sum_{q=1}^{{n}_{D}}\left({\left|{h}_{D}^{s,q}\right|}_{1}\right),\forall{p}\in\left(s+1,\dots,s+40\right)$$

30 $${C}_{\mu}=\raisebox{1ex}{${\mu}_{B}-{\mu}_{D}$}\!\left/\!\raisebox{-1ex}{${n}_{B}+{n}_{D}$}\right.$$

31 $${C}_{\sigma}=\raisebox{1ex}{$\sum{({h}_{B}^{s}-{\mu}_{B})}^{2}-\sum{({h}_{D}^{s}-{\mu}_{D})}^{2}$}\!\left/\!\raisebox{-1ex}{$\left({n}_{B}+{n}_{D}\right)$}\right.$$

Thus, the control and diseased samples are separated into respective clusters. The terms in ${C}_{\mu}$ and ${C}_{\sigma}$ (30) and (31) for both the control and diseased class are used to understand changes, thus coefficients and corresponding basis vectors grouped into four matrices$D$,$B$, $P$ and $C$ over time, aiding in the classification of clusters based on these metrics.

Finally, the relative expression are computed as^11^:

32 $${X}_{r}={\gamma}_{1}{\left[\frac{{d}_{ij}}{{b}_{ij}}\right]}_{gxs}\times{\gamma}_{2}{\left[\frac{{p}_{ij}}{{b}_{ij}}\right]}_{gxs}\times{\gamma}_{3}{\left[\frac{{{p}^{c}}_{ij}}{{b}_{ij}}\right]}_{gxs}$$

For details about variational analysis and biomarker ranking, please follow^11^.

#### Convergence and stability analysis

**Fig. 2:**
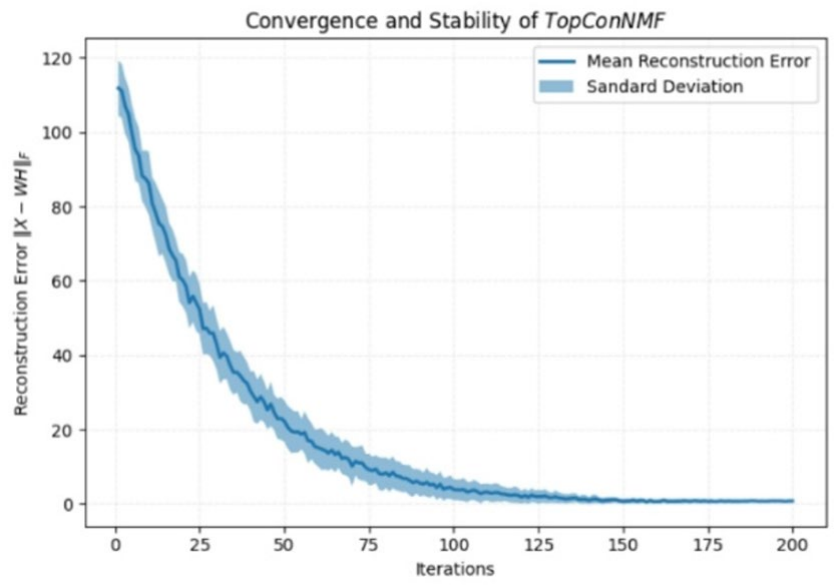
Convergence and Stability of *TopConNMF*.

In this study, the convergence and numerical stability of the proposed *TopConNMF* framework were examined primarily through extensive empirical validation. To assess the robustness of the decomposition with respect to initialization, the model was rigorously simulated more than 60 independent times on each dataset, with randomly generated initial matrices W and H in every run. For each iteration across these repeated experiments, the reconstruction error was recorded, and the corresponding mean and variance were computed. As illustrated in Fig. 2, the mean reconstruction error decreases smoothly with iterations and stabilizes after approximately 150 iterations, while the variance across runs remains extremely small, indicating highly consistent convergence behavior. This empirical evidence demonstrates that the proposed optimization process is stable, initialization-resilient, and reliably convergent across both datasets.

## Results and discussion

### Data description

The proposed is evaluated on two real-world datasets. The first dataset^11,25^, includes gene expression data Type 2 Diabetes Mellitus (T2DM) exposed population. The dataset encompasses samples from the gastrocnemius muscles of 100 rats. Out of the entire population, half of the population were subjected high-fat diet, while the other half was fed with a standard diet. Further, the data were collected at 4-week intervals, starting from 4 to 20 weeks of age. This dataset is comprehensive, containing 16,734 genes.

The second dataset^11,25^, is composed of miRNA data related to Huntington’s disease (HD). The expression profiles were collected from the stratum tissue of rats exposed to HD from 2 to 10 weeks of age, with data collection occurring every 4 weeks. This dataset was gathered from both wild rats and knock-in mice with varying CAG repeat lengths of 20 and 80 and four replications were performed at each time point. A collection of 1626 miRNA expressions were used in this study.

### Metrics for evaluation

To show how well the proposed method works, we need to do two analyses. First, we must assess how efficiently *TopConNMF* can decompose the data. Second, we should compare it with the *state-of-the-arts*. We measure the decompose capability of *TopConNMF* using several metrics: reconstruction error (RE), structural similarity index (SSI), root mean squared difference (RMSD), normalized mutual information (NMI), and correlation coefficient (CC)^12,27^. For comparing how well the framework predicts disease-related biomarkers, we use two metrics: the area under the receiver operating characteristic curve (AUC) and the area under the precision-recall curve (AUPR). The AUC metric effectively summarizes model performance over various thresholds without being affected by scale or classification bias, while the AUPR offers detailed insights, particularly useful for imbalanced datasets, together giving a thorough assessment of the model’s predictive power^25^. An AUC value close to 1 shows that the method is good at separating classes. Similarly, an AUPR value near 1 means the classifier is effective, showing a good balance between precision and recall^28^.

### Parameter setting and ablation study

#### Selecting optimal regularization coefficient

**Fig. 3:**
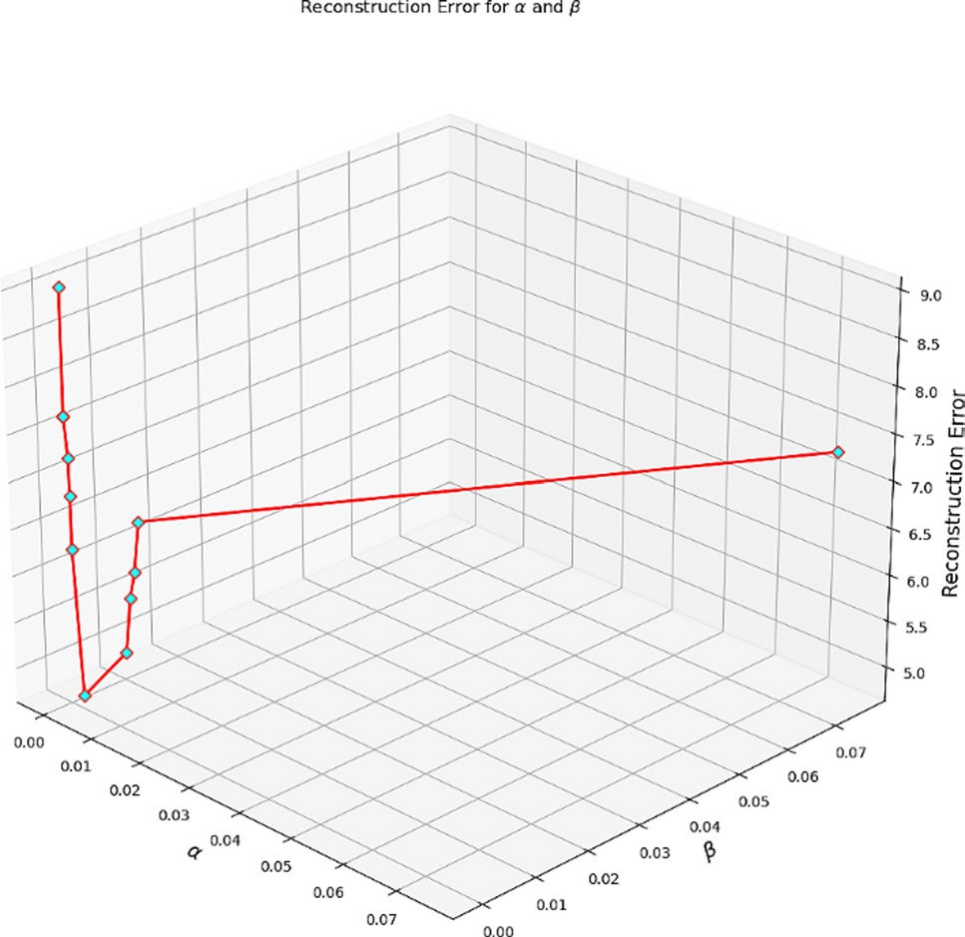
Graphical analysis of the change in reconstruction error (RE) with respect to the regularizing coefficients.

The regularization parameters in (13) determine the strength of the constraints applied to both the basis and coefficient matrices, enabling the incorporation of domain knowledge into the adaptation process. When prior information regarding feature relevance is available, assigning appropriate weights helps guide the model toward more meaningful and generalizable representations. Therefore, careful selection of these parameters is essential. In this work, we employed a structured grid search procedure, supported by literature evidence^11,25^, to explore a range of candidate values for the regularizers. As illustrated in Fig. 3, this analysis revealed that the reconstruction error reaches its optimum at $\lambda=0.0150$ and $\beta=0.0095$, which were consequently adopted as the final parameter settings.

#### Reconstruction performance of *TopConNMF*

The proposed computational method is inherently randomly initialized, leading to varying performance outcomes based on different pre-set factorization ranks ($k$). Thus, to gain insight into the average performance of the proposed method, it was simulated 30 times for each pre-set$k\in\{19,20,21,22,23\}$^11,28^. The mean (µ) and standard deviation (σ) were calculated using four different metrics namely: reconstruction error (RE), structural similarity index (SSI), normalized mutual Information (NMI), and correlation coefficient (CC) to understand the reconstruct performance of the proposed method^12^. It is also worth mentioning that just to understand the decomposition capability of the proposed method, it was tested on six benchmark time-invariant ‘omic datasets^5,11,12^: colon, prostate, GLI-85, Leukemia, Tox_171, and Lymphoma, and then on two time varying ‘omic datasets (i.e. T2DM and HD). An overall reconstruction performance for all eight datasets is presented in Table 1. Further, as the study strictly focuses on time-varying, ‘omic data, a comparative analysis was conducted with the *state-of-the-art* methods on the two time varying ‘omic data namely, T2DM and HD datasets and the results are presented in Table 2.

#### Comparative study

As stated earlier the proposed method in hand is holds random initialization, leading to different outcomes when applied on the complex biomarker data. Thus, to gauge its average efficacy, the proposed framework was simulated 30 times for each distinct biomarker set, with the resulting mean and standard deviation computed using six different benchmark classification techniques^11,25^. The classification performance in terms the area under the curve (AUC) for both T2DM and HD datasets, are detailed in Table 3. While Table 4 lists the area under the precision-recall curve (AUPR) for the same datasets.

When the performance of the method was measured against six other classifiers^11,36^ using the top 3% of biomarkers associated with the diseases, it was noted that our method generally exceeded the performance of other advanced methods, especially for$k=21$, with CART as a classifier.

**Table 1:**
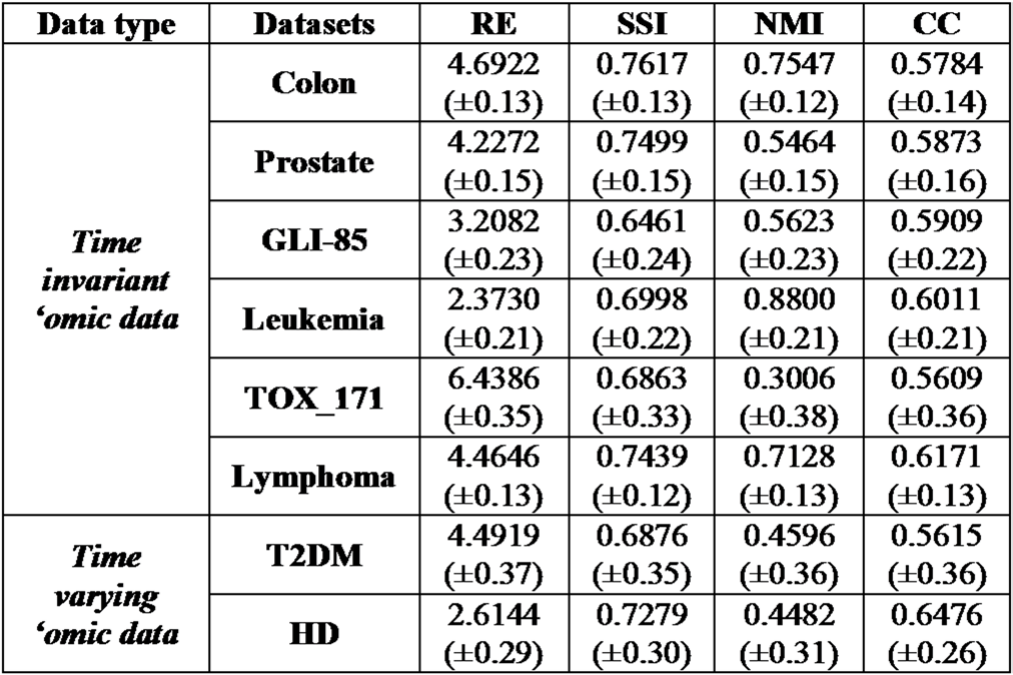
Average reconstruction performance of the proposed method (µ ± σ).

**Table 2:**
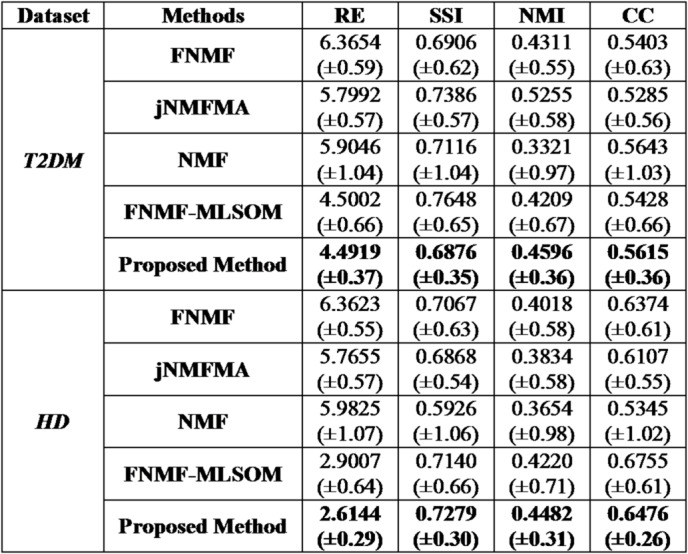
A comparative analysis of the reconstruction performance of the proposed method with other state of the arts (µ ± σ).

Consequently, further research was conducted using the aforementioned parameters and classifier. Table 3 offers a comparative analysis of our findings with other leading methods for the T2DM and Huntington’s disease datasets.

Moreover, Table 5 highlights the superior performance of our method over other *state-of-the-arts*, particularly as the number of biomarkers linked to the disease increases. A noticeable decrease in AUC and AUPR values was observed among all advanced methods, attributed to the rise in the number of biomarkers, which introduces significant noise into the gene/protein expressions driven by the disease, thereby diminishing the predictive accuracy.

Nonetheless, our proposed method distinguishes itself by showing a marked decrease in variability and a notable improvement in the classification performance metrics.

**Table 3:**
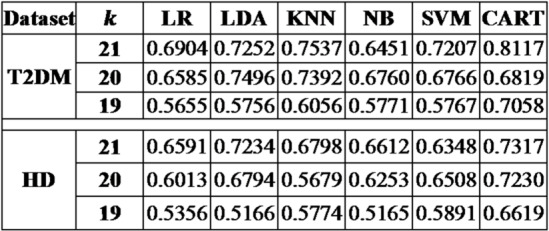
Average AUC value for the proposed method.

**Table 4:**
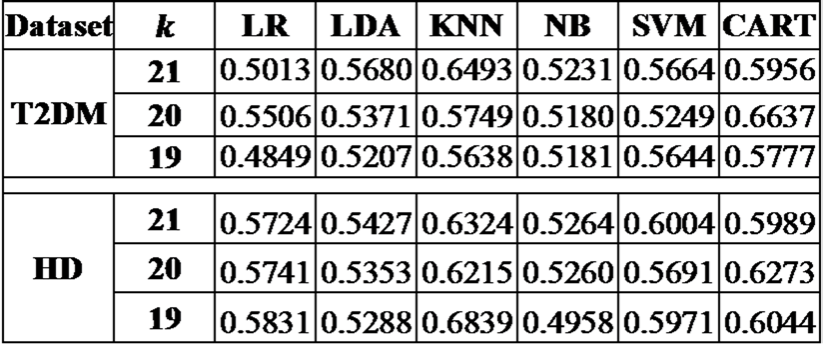
Average AUPR value for the proposed method.

#### Statistical validation

To statistically validate the observed performance differences, Welch’s t-test was conducted based on the reported mean $\pm$ standard deviation over 30 independent simulations. The proposed method demonstrated statistically significant improvement in reconstruction error for the HD dataset (t = − 2.232, *p* = 0.031, Cohen’s d = 0.576), indicating a moderate effect size. For the T2DM dataset and across clustering quality metrics (SSI, NMI, CC), no statistically significant differences were observed ($p>0.05$), suggesting comparable performance to the strongest baseline. Additionally, comparisons with classical NMF-based methods revealed statistically significant improvements in predictive performance metrics, further confirming the robustness and effectiveness of the proposed framework.

#### Biological relevance

Most of the biomarkers identified through our proposed gene selection strategy demonstrate a significant relationship with pathways implicated in T2DM exposure. Several genes have been validated to support our findings, and their frequency of occurrence is summarized in Table 6. Notably, HMGCS2, a mitochondrial enzyme, is a hub gene regulating diabetic retinopathy.

**Table 5:**
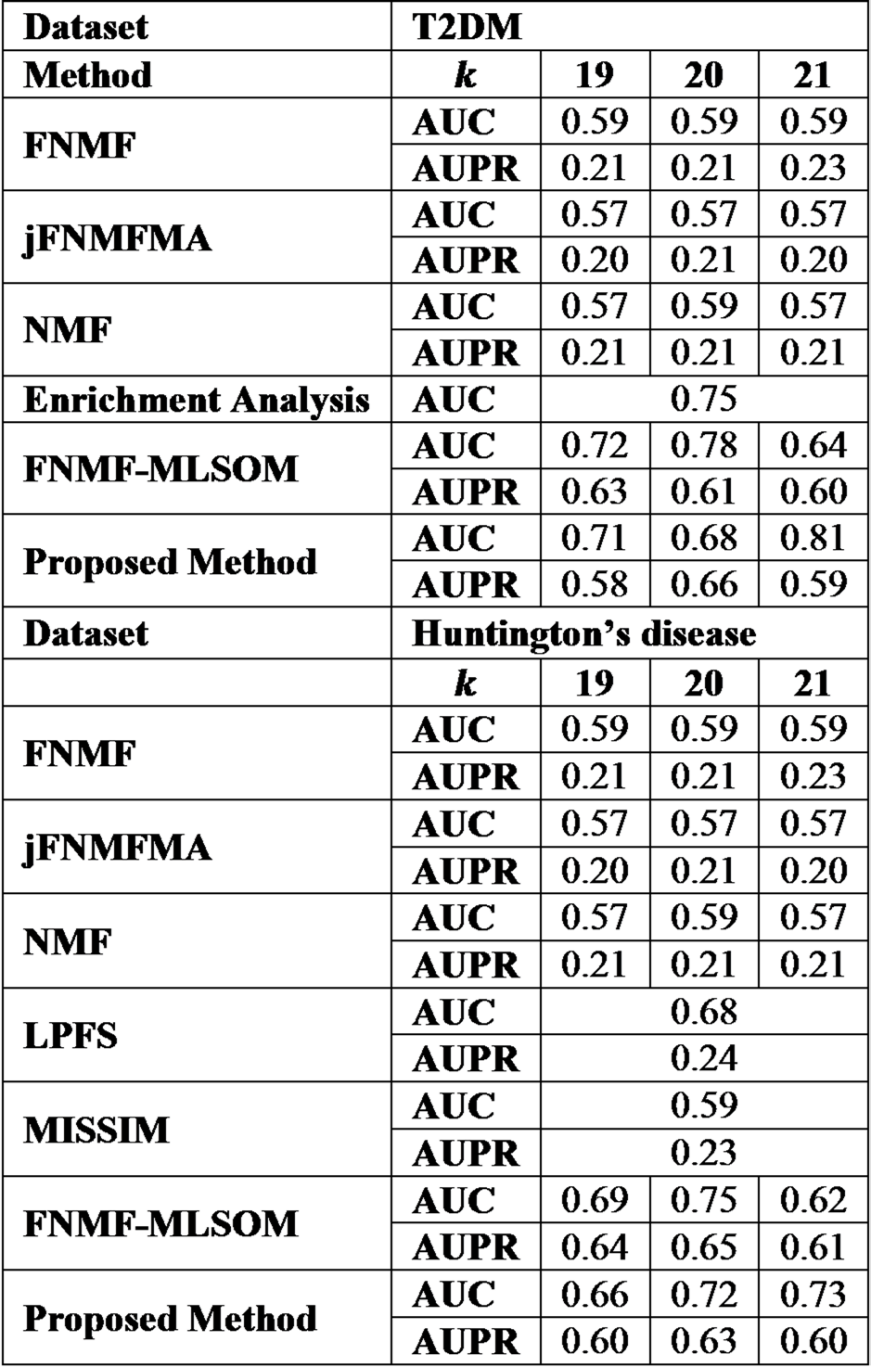
Comparative study on time varying ‘omic data.

In diabetic mice, upregulation of HMGCS2 has been observed, and its deficiency is linked to errors in ketogenesis and non-alcoholic fatty liver disease^37–39^.Furthermore, ACOT1 and PDK4 are crucial genes related to T2DM. ACOT1, known for hydrolyzing long-chain acyl- CoAs, is associated with triglyceride levels, fatty acid oxidation, mitochondrial function, and insulin signaling^40–42^. PDK4 is identified as a hub gene for diabetic cardiomyopathy, linked to vascular calcification and autophagy^41^. These genes, among others, provide substantial insights into the molecular mechanisms underlying T2DM.

Although, in the study of Huntington’s disease, understanding miRNA profiles is essential but difficult due to their intricate impact on genes and the disease’s development, with different miRNAs influencing various stages of the disease. Therefore, to validate these miRNA profiles biological pathway analysis is the most prominent choice, but authors deliberately choose not to focus in this study. For more detailed analysis, readers should refer^43–45^.

**Table 6:**
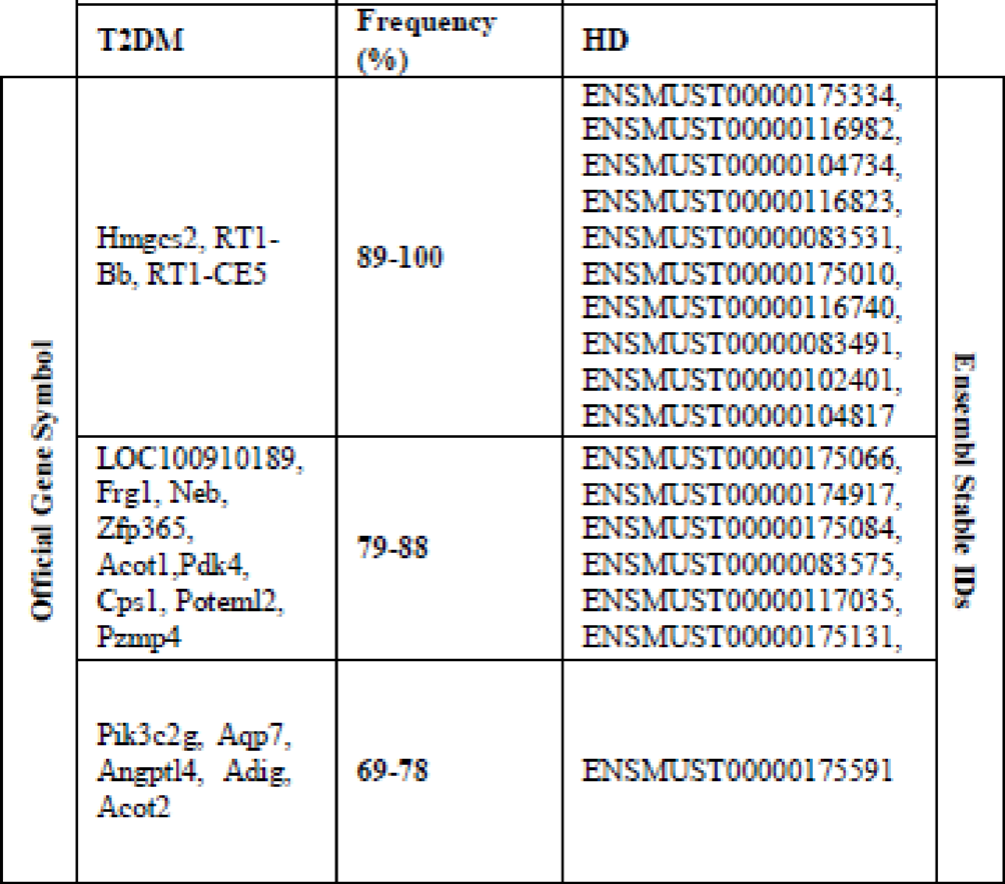
Most frequently occurred ‘omic biomarkers.

## Conclusion

The study presents *TopConNMF*, a novel nonnegative matrix factorization based biomarker identification paradigm designed for identifying differentially expressing ‘omic markers for better disease surveillance. This model integrates a dual topological-sparsity regularization to account for the manifold structures within high-dimensional data thus enhancing genetic discernibility while identifying the disease developing biomarkers. Our validation on both T2DM and HD time-varying datasets demonstrated superior performance in gene selection and classification performance, confirming its efficacy in compressing data dimensions.

Future research will address current limitations, such as sensitivity to outliers, by exploring alternative loss functions and incorporating graph theories to enhance robustness and predict novel disease associations.

## Data Availability

The datasets analyzed during the current study are available in the NCBI-GEO repository, link: [https://www.ncbi.nlm.nih.gov/geo/]. Further, details regarding accessing the data and the implemented code can be obtained from the corresponding author (A. Dey, email: [anirbandey@soa.ac.in]) upon reasonable request.
